# Dosage de la 25 OH vitamine D: expérience du laboratoire central de biochimie clinique du Centre Hospitalier Ibn Sina

**DOI:** 10.11604/pamj.2014.17.152.3341

**Published:** 2014-03-03

**Authors:** Najat Handor, Sanae Elalami, Mounya Bouabdellah, Abdelatif Srifi, Hicham Esselmani, Laila Benchekroun, Layachi Chabraoui

**Affiliations:** 1 Laboratoire Central de Biochimie Clinique, Centre hospitalier Ibn Sina (CHIS), Rabat, Maroc

**Keywords:** Vitamine D, dosage, laboratoire, biochimie, Vitamine D, dosage, laboratory, biochimistry

## Abstract

La connaissance de la physiologie de la vitamine D a considérablement progressé ces dernières années, la faisant passer de simple vitamine à tropisme purement phosphocalcique et osseux à celui d'hormone jouant un rôle crucial dans de nombreux mécanismes physiologiques et dont le déficit est impliqué dans plusieurs pathologies. Nous présentons, dans ce travail, l'expérience du laboratoire central de biochimie dans le dosage de la 25 OH vitamine D. Enquête descriptive exhaustive portant sur les dosages de la 25 OH vitamine D effectués chez 350 patients du Centre Hospitalier Ibn Sina (CHIS). La méthode adoptée est un dosage immunologique par chimiluminescence sur microparticules réalisés sur l'auto-analyseur ARCHITECT 8200 (ABBOTT^®^) pendant une période de six mois (du 01 Juin 2011 au 31 Decembre 2011). Quatre vingt et onze pourcent des patients présentent une hypovitaminose. En effet 76,6% des patients souffrent d'une insuffisance en vitamine D, 12,3% de carence vitaminique et 2,6% d'ostéomalacie. L'hypovitaminose est associée dans 92,18% des cas à une normocalcémie, dans 76,87% des cas à une Hyperparathormone, dans 92,81% à des troubles thyroïdiens et dans 97,5% à une insuffisance rénale. Par ailleurs aucune relation statistiquement significative n'est établie entre l'hypovitaminose et le diabète. A la lumière des implications des hypovitaminoses dans plusieurs pathologies ou dans leurs complications et au vu du nombre élevé de patients présentant un déficit en vitamine D, il paraît judicieux d'envisager une étude épidémiologique sur le statut en vitamine D dans la population marocaine comme outil préventif avant d'élargir le dosage de ce marqueur biologique en vue d'une éventuelle supplémentation.

## Introduction

La connaissance de la physiologie de la vitamine D a considérablement progressé ces dernières années, la faisant passer de simple vitamine à tropisme purement phosphocalcique et osseux à celui d'hormone jouant un rôle crucial dans de nombreux mécanismes physiologiques et dont le déficit est impliqué dans plusieurs pathologies. Nous présentons, dans ce travail, l'expérience du laboratoire central de biochimie dans le dosage de la 25 OH vitamine D.

## Méthodes

Enquête descriptive exhaustive portant sur les dosages de la 25 OH vitamine D effectués chez 350 patients du centre hospitalier Ibn Sina de Rabat (CHIS). La méthode adoptée est un dosage Enquête descriptive exhaustive portant sur les dosages de la 25 OH vitamine D effectués chez 350 patients du centre hospitalier Ibn Sina de Rabat (CHIS). La méthode adoptée est un dosage immunologique par chimiluminescence sur microparticules réalisés sur l'auto-analyseur ARCHITECT 8200 (ABBOTT^®^) sur une période de six mois, effectuée au laboratoire central de biochimie du CHIS de Rabat, s’étalant du 1er juin 2011 au 31 décembre 2011.

**Analyse statistique**: Une analyse descriptive des données a été effectuée. Les variables qualitatives ont été exprimées en effectif et pourcentage. Pour la comparaison entre l'hypovitaminose et d'autres paramètres biologiques, nous avons utilisé le test de khi 2 et en cas de besoin, le test exact bilatéral de Fisher pour la comparaison de pourcentages. Le degré de signification p a été fixé à 5% et les données ont été saisies et analysé à l′aide du logiciel SPSS (version 19.0).

## Résultats

**Figure 1:**
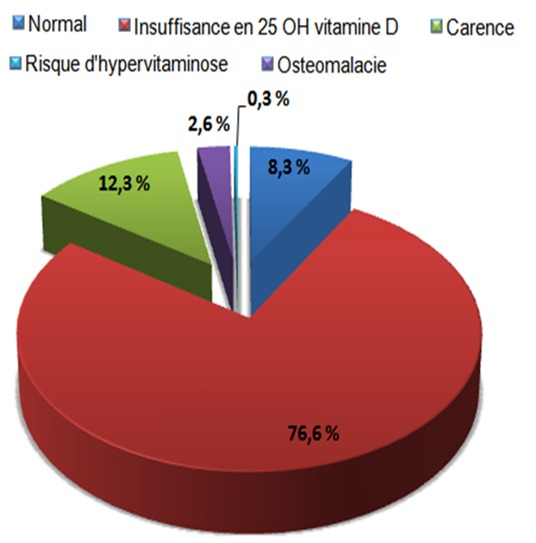
Répartition de la population étudiée en fonction du statut en 25 OH vitamine D

Au cours de la période d′étude de 350 patients ont bénéficié d′un dosage de la 25 OH vitamine D avec un sex-ratio H / F de 0,27. Quatre-vingt et onze pour cent de notre population présentait une hypovitaminose (320/350). En effet 76,6% (268/350) des patients souffraient d′insuffisance en vitamine D, 12,3% (43/350) de carence en vitamine D et de 2,6% (9/350) de l′ostéomalacie (Figure 1). Le Tableau 1 illustre la relation entre l'hypovitaminose et d'autres paramètres biologiques: ainsi l'hypovitaminose est associée dans 92,18% (295/320) des cas à une normocalcémie, dans 76,87% (246/320) des cas à une hyperparathyroïdie, dans 92,81% (297/320) à des troubles thyroïdiens et dans 97,5% (312/320) à une insuffisance rénale. Par ailleurs aucune relation statistiquement significative n'a été établie entre l′hypovitaminose et le diabète.

**Tableau 1:** Paramètres biologiques étudiés en fonction l'hypovitaminose (25OH vitamine D)

	Hypovitaminose n=320		p
	n	%	
Normocalcémie	295	92.18	0.001
Hyperparathormone	246	76.87	0.004
Diabète	1	0.31	NS*
Troubles thyroïdiens	297	92.81	0.001
Insuffisance rénale	312	97.5	<0.001

## Discussion

L’évaluation du statut en vitamine D se fait au moyen du dosage de la 25 OH vitamine D qui est considérée comme un bon témoin des réserves en vitamine D. Le taux est actuellement fixé à 30 ng/ml au minimum, jusqu’à 70 ng/ml au maximum [1]. Une subcarence ou une carence sont présentes chez la moitié de la population adulte, la prévalence augmentant avec l′âge et chez les femmes [1]. Ces données concordent avec nos résultats qui mettent en évidence un pourcentage de 91,4% d'hypovitaminose (Figure 1) avec un sexe ratio H/F de 0,27. Le rôle le plus important de la vitamine D est le maintien de l′homéostasie phospho-calcique par augmentation de l′absorption intestinale du calcium et du phosphore, et la promotion de la minéralisation osseuse. La conséquence d′un déficit en vitamine D est une diminution de l′absorption intestinale du calcium et une tendance hypocalcémique, induisant une augmentation de la concentration de parathormone (PTH) qui stimule le remodelage osseux normalisant ainsi la calcémie [2]. Ce mécanisme explique les pourcentages de 92,18% de la normocalcémie et de 76,87% de l'Hyperparathormone retrouvés dans les cas d′hypovitaminoses de notre série (Tableau 1). Aussi il est à noter que de nombreuses études ont évalué la relation entre les concentrations de 25OHD et les concentrations de la PTH dans des populations apparemment en bonne santé en cherchant à définir la concentration de 25OHD au dessous de laquelle la PTH peut augmenter [3]. La grande majorité de ces études révèle une relation statistique négative entre ces deux paramètres. Cette relation n’était toutefois en général pas linéaire: la PTH diminuait jusqu’à ce que la 25OHD atteigne une valeur seuil au-dessus de laquelle la PTH est stable. Ces études ont été récemment listées et commentées par Aloia et al [3].

Dans notre série et comme le rapportent d'autres auteurs [2] nous n'avons pas trouvé de seuil de 25OHD au-dessus duquel la PTH ne diminuait plus cela pourrait être du au choix de notre population. L'hypothèse du rôle d'une carence en vitamine D en tant que facteur de risque de diabète est née de nombreuses constatations épidémiologiques et de données expérimentales. La cellule β-pancréatique exprime le récepteur de la vitamine D et possède une activité 1 a-hydroxylase. En régulant la concentration calcique extracellulaire et les flux calciques transmembranaires, la vitamine D contribue à moduler la sécrétion d'insuline et la sensibilité à l'insuline [4]. Elle pourrait aussi améliorer la sensibilité à l'insuline et favoriser la survie des cellules β-pancréatiques en les protégeant de l'apoptose et en modulant la production et les effets des cytokines pro-inflammatoires qui sont à l'origine de l’état d'inflammation présent dans le diabète de type 2 [4].

Chez le sexe masculin les résultats de trois études révèlent une association significative entre les concentrations élevées de vitamine D et un faible risque de diabète de type 2. Par ailleurs une telle association n'est retrouvée chez le sexe féminin que dans une étude de Women's Health Study [5]. Quant à notre série nous ne retrouvons pas de relation entre l'hypovitaminose et le diabète cela pourrait être expliqué par le choix de notre population. Quatre-vingt-douze pour cent des troubles thyroïdiens retrouvés dans notre série sont associés à une hypovitaminose (Tableau 1). Plusieurs études soulignent la relation entre la carence en vitamine D et les maladies thyroïdiennes. Ceci peut être expliqué par la liaison de la vitamine D à des récepteurs similaires à ceux des hormones thyroïdiennes appelés steroid hormone receptors. Aussi il a été démontré qu'une atteinte du gène codant pour le récepteur de la vitamine D prédisposait aux maladies thyroïdiennes auto-immunes comme la maladie de Basedow et la thyroïdite de Hashimoto [6]. Pour ces raisons, il est important pour les patients atteints de troubles thyroïdiens de comprendre le système de fonctionnement de la vitamine D.

Au niveau rénale la 25 OH D est transformée en 1,25 OH 2 D, métabolite le plus actif de la vitamine D. Sa synthèse a lieu principalement dans le rein en condition physiologique plus précisément dans la membrane interne des mitochondries des cellules des tubules contournés proximaux. La 1-alpha-hydroxylation de la 25 OH D est réalisée par la 25 OH D-1-alphahydroxylase, complexe enzymatique incluant un cytochrome p450 spécifique (CYP27B1) [7]. La relation entre l'attente rénale et le déficit en vitamine D semble donc évidente ce qui explique le pourcentage de 97,7% des hypovitaminoses chez les insuffisants rénaux retrouvé dans notre série (Tableau 1).

## Conclusion

Au-delà d'un rôle bien démontré dans le métabolisme ostéo-musculaire, la vitamine D semble être un paramètre important de santé en général. De nombreuses données épidémiologiques corroborées par un rationnel théorique suggèrent qu'un déficit en vitamine D, particulièrement fréquent dans la population générale, pourrait constituer un facteur de risque du diabète, de maladies cardiovasculaires, immunitaires Des études d'intervention randomisées de grande envergure restent à entreprendre pour démontrer avec certitude la place de la vitamine D dans la prévention ces pathologies. D'ores et déjà, une politique de supplémentation vitaminique paraît légitime dans le but de maintenir un état de santé optimal chez tous les sujets présentant une subcarence en vitamine D.
